# Coating Reactions on Vanadium and V-Si-B Alloys during Powder Pack-Cementation

**DOI:** 10.3390/ma13184099

**Published:** 2020-09-15

**Authors:** Georg Hasemann, Chad Harris, Manja Krüger, John H. Perepezko

**Affiliations:** 1 Institute of Materials and Joining Technology, Otto-von-Guericke University Magdeburg, Universitätsplatz 2, 39106 Magdeburg, Germany; manja.krueger@ovgu.de; 2 Department of Materials Science and Engineering, University of Wisconsin-Madison,1509 University Ave., Madison, WI 53706, USA; charris9@wisc.edu (C.H.); perepezk@engr.wisc.edu (J.H.P.)

**Keywords:** refractory metals, coatings, pack-cementation, oxidation protection

## Abstract

Alloys in the V-Si-B system are a new and promising class of light-weight refractory metal materials for high temperature applications. Presently, the main attention is focused on three-phase alloy compositions that consist of a vanadium solid solution phase and the two intermetallic phases V_3_Si and V_5_SiB_2_. Similar to other refractory metal alloys, a major drawback is the poor oxidation resistance. In this study, initial pack-cementation experiments were performed on commercially available pure vanadium and a three-phase alloy V-9Si-5B to achieve an oxidation protection for this new type of high temperature material. This advance in oxidation resistance now enables the attractive mechanical properties of V-Si-B alloys to be used for high temperature structural applications.

## 1. Introduction

In a previous study on the phase stability in the Mo-Si-B system it was determined that other refractory metals such as Nb, W and V are completely soluble not only in the Mo_SS_ (solid solution) phase but also in the Mo_5_Si_3_ (T_1_) and the Mo_5_SiB_2_, (T_2_) phase [1]. Both W and Nb destabilize the Mo_3_Si phase, but V is quite soluble in this phase. Thus, the addition of V to Mo-Si-B alloys can retain the three phase Mo_SS_ + Mo_3_Si + T_2_ microstructure. Moreover, the substitution of V for Mo will result in a considerable reduction in alloy density since the density of Mo is 10.2 gm/cm^3^ while that for V is only 5.96 gm/cm^3^ [2]. Moreover, with complete substitution V-Si-B alloys exhibit excellent high temperature creep resistance that is comparable to that for Ni base superalloys [3,4] as well as a plastic strain of 8% under 1500 MPa at room temperature [5]. Vanadium-based alloys are discussed as structural materials for fusion reactors. Especially V-Cr-Ti solid solution alloys were identified as material for liquid lithium-cooled blankets [6,7,8,9]. At the same time, the oxidation resistance of V, like that of other refractory metals, is poor.

For Mo-Si-B alloys after an initial transient period where the loss of Mo by MoO_3_ volatilization above about 700 °C enriches the surface in B and Si a protective layer of borosilica glass forms at the surface [10]. The oxidation rate is decreased due to the inhibition of oxygen transport through the borosilica scale, but the rate is still too high for applications with long lifetimes. However, coatings have been developed to enhance the environmental resistance and lifetime [11]. For the V-Si-B alloys the oxidation of V yields V_2_O_5_ which is a liquid above 700 °C. While the V_2_O_5_ does exhibit some evaporation at high temperatures, it remains present and acts to disrupt the development of a continuous surface scale and can promote cristobalite formation that prevents the development of any effective intrinsic oxidation resistance [12]. Additions of Cr, Ti, Al, Si and B can improve the oxidation behavior of vanadium alloys, but the modified alloys still need a suitable oxidation protection [7,12,13].

Therefore, a coating will be necessary to survive harsh operation conditions of aggressive environments without degradation in order to realize any benefits from V addition to Mo-Si-B alloys, but also for newly developed V-Si-B alloys [3,5,14,15,16].

Besides other strategies to obtain oxidation resistant coating by using pre-ceramic polymers [16,17,18] and PVD techniques like magnetron sputtering [19,20,21], pack cementation by co-deposition of Si and B seems to be one of the most promising coating strategies to protect refractory based metals and alloys from oxidation and corrosion by water vapor or molten salts [22,23,24].

In order to develop a coating procedure a two-step process has been adopted with the first step involving the deposition of a Mo coating on the substrate that is followed by a pack cementation co-deposition of Si and B [23]. The final step is the conditioning treatment to develop the full coating microstructure.

## 2. Materials and Methods

Two different sets of substrate materials were chosen and prepared for coating, pure vanadium and a V-9Si-5B alloy. In general, the pack cementation process involved a two-step procedure: (1) Depositing a Mo layer on the outer surface of the pure vanadium and V-Si-B substrate and (2) a co-deposition of Si and B via a powder pack treatment. The details on coating the two V and V-Si-B substrate materials will be given in the following section.

### 2.1. Pure Vanadium Substrate

Samples were cut from 99.5% commercially pure 0.5-inch diameter vanadium bar stock approximately 2 mm thick. Sample edges were rounded to reduce the risk of sample failure due to stress cracking of the coating by stress concentrations at sample edges. All surfaces were polished using up to 320 grit SiC paper.

The samples were coated in a Mo pack cementation process. The pack ingredients were as follows: 40 wt.% pure commercial Mo powder with a particle size less than 50 µm as raw material, 3 wt.% NH_4_Cl as activator, and 57 wt.% Al_2_O_3_ as filler material. After weighing and mixing, the material was ground in a mortar and pestle for 30 min. Samples were buried and gently compressed in intimate contact with approximately 15 g of powder mixture in an alumina boat. The alumina boat was sealed in an alumina tube, evacuated of air and purged with Ar gas (99.9% purity). The tube was placed in a furnace set to 1000 °C and run for 10 hrs under Ar flow of approximately 25 mL/min.

The samples were then coated by another pack cementation process which promotes diffusion via gaseous transport of Si and B into the Mo layer by a NaF activator and a powder mixture with a Si:B ratio of 35:1. Samples were buried and gently compressed in intimate contact with approximately 10 g of powder mixture in an alumina crucible. The alumina crucible was sealed in an alumina tube, evacuated of air and purged with Ar gas (99.9% purity). The tube was placed in a furnace set to 1000 °C and run for 50 h under Ar flow of approximately 25 mL/min.

### 2.2. V-9Si-5B Alloy Substrate

The alloy V-9Si-5B (at.%) is a hypo- eutectic ternary alloy which is composed of vanadium solid solution (V_SS_) dendrites along with a V_SS_-V_3_Si-V_5_SiB_2_ ternary eutectic microstructure [25] and is an attractive alloy for developing V-based V-Si-B alloys for structural applications due to its room temperature mechanical properties [5].

The alloy was cast via levitation melting into 95 mm long rods with a diameter of 8 mm. After casting cuboidal samples (3 × 3 × 5 mm in geometry) were cut by electrical-discharge machining (EDM). The sample edges were rounded to avoid cracking by stress concentrations and were also ground using 320 grit SiC paper to increase the surface roughness.

Molybdenum was deposited via a spray process using a slurry of Mo powder with a particle size between 1 and 5 µm and a transparent lacquer (Behlen 11-B61406, Behlen Ltd. Europe, Edinburgh, UK) in a ratio 1:10 mL. The slurry was filled in an airbrush gun and the samples were coated 6-10 times. After each spray coat, the samples were set to dry for 20–30 min and placed on an alumina boat and quartz glass. The layers were pyrolyzed and sintered stepwise after each spray coat at 1300 °C in vacuum (10^−4^ bar or better) using a heating rate of 13 K/min, a dwell time of 2 h and a cooling rate of 7 K/min.

In a second step, Si and B were co-deposited onto the Mo coated V-9Si-5B alloys through pack cementation using a powder with a Si and B weight percent ratio of 35:1 under an Ar atmosphere. Samples were buried and gently compressed in intimate contact with approximately 15 g of powder mixture in an alumina crucible. The alumina crucible was sealed in an alumina tube, evacuated, and purged with Ar gas. The tube was placed in a furnace set to 1000 °C and run for 50 h under an Ar flow of approximately 25 mL/min.

### 2.3. Conditioning of Packed Samples

**Figure 1:**
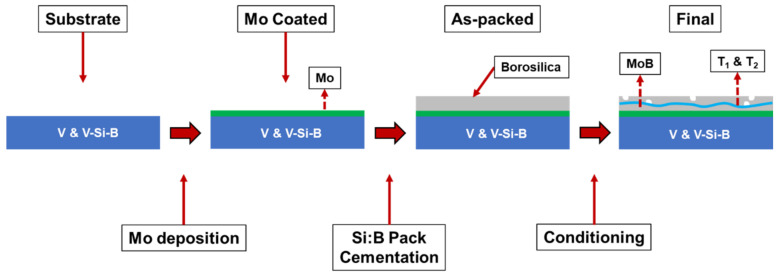
Schematic illustration of the coating process for pure vanadium and V-Si-B substrates.

After pack cementation, some samples were then conditioned at temperatures between 1100 °C and 1300 °C in dry air. The conditioning process anneals the coated sample in ambient atmosphere, allowing for simultaneous production of the protective borosilicate layer and the T_1_ and T_2_ phases by solid state reaction of MoSi_2_, MoB, and Mo. Figure 1 shows the evolution of samples from as-cut coins through conditioning.

### 2.4. Metallographic Preparation

In order to study the microstructure pack-cementation and conditioning, samples were hot mounted (Struers Poly Fast, Struers GmbH, Willich, Germany) and ground from 180 grit down to 2000 grit, followed by mechanical polishing with a 3 μm and 1 μm diamond suspension and finished using colloidal silica. The microstructures were investigated using a Zeiss Merlin or Zeiss Supra 50 VP (Carl Zeiss Microscopy, Oberkohen, Germany) scanning electron microscope (SEM). SEM images were typically performed in the backscattered electron (BSE) mode. To determining the diffusion path, electron backscatter diffraction (EBSD) measurements (Oxford Instruments, Bognor Rigis, UK) were performed for phase identification after conditioning. For EBSD, electrons were accelerated using 15 kV and a grit of 517 × 376 pixels with as step size of 0.11 µm was used. For phase identification, the following crystallographic parameters and databases has been used, Table 1.

## 3. Results and Discussion

**Figure 2:**
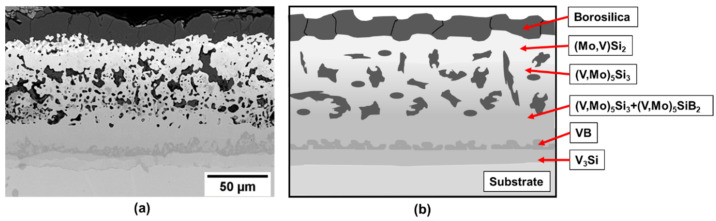
(**a**) Coating structure on pure vanadium substrate after exposure at 1100 °C for 10 h and (**b**) schematic illustration of the coating structure.

As an initial approach samples of pure vanadium were processed by the two-step method. The cross-section image of the coated sample after exposure at 1100 °C for 10 h is presented in Figure 2a). EBSD (electron backscatter diffraction) has been used for phase identification. The resulting microstructure is depicted schematically in Figure 2b). The 10 h at 1100 C is the time needed to fully develop the multiphase multilayer coating structure and the exposure in air demonstrates the effective oxidation protection of the coating. For the analysis of phase evolution in the coating after exposure, the construction of the diffusion pathway provides useful insight.

**Figure 3:**
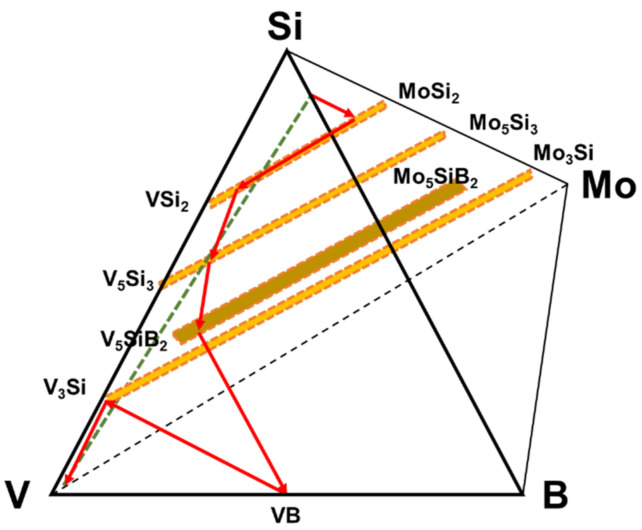
Diffusion pathway of the coating structure applied on pure vanadium plotted on the quaternary isotherm at 1100 °C.

The diffusion pathway associated with the phase evolution is plotted in the V-Si-B-Mo quaternary diagram that is shown by involving only the relevant phases in Figure 3.

From the diffusion path it is evident that the initial reactions starting from the Si-B edge involve silicide phase formation of (Mo, V)Si_2_ that is enriched in V to (V, Mo)Si_2_, followed by (V, Mo)_5_Si_3_ and (V, Mo)_5_SiB_2_. Even though the disilicide and T_1_ phases have limited B solubility, they do not appear to impede the penetration of B as evidenced by the VB layer that forms adjacent to the substrate. Since the disilicide and (V, Mo)_5_Si_3_ phases lie above the dotted mass conservation line between the Si-B edge and the pure V corner, the diffusion path must cross this line as demonstrated by the formation of the (V, Mo)_5_SiB_2_ and VB phases. It is also important to note that there is no evidence for a V_2_O_5_ oxide phase which could disrupt the coating and impair the oxidation resistance. This confirms the effectiveness of the amorphous borosilica surface layer as a barrier to oxygen transport.

**Figure 4:**
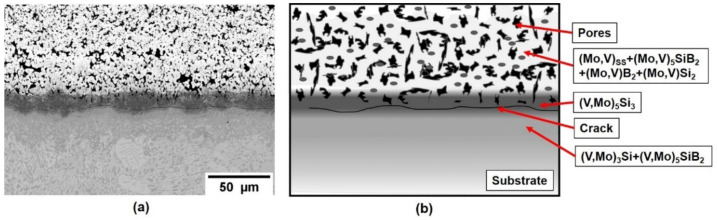
(**a**) Coating structure on a V-9Si-5B alloy after pack cementation at 1000 °C for 50 h in flowing Ar and (**b**) schematic illustration of the as-packed structure.

Based upon this initial success on pure vanadium, a V-Si-B three-phase V_SS_ + V_3_Si + V_5_SiB_2_ alloy, V-9Si-5B, was treated in the next step. The initial as-packed state of the coating is shown in Figure 4, showing the successful implementation of Si and B into the Mo spray coat and the formation of Mo-based silicides and borides. The pores are likely a result of incomplete sintering of the Mo spray coating and/or volume changes during the phase conversation upon conditioning. Between substrate and the silicide- and boride-phases a (V, Mo)_5_Si_3_ and cracks have formed. The crack formation is likely related to the mismatch in the coefficient of expansion between the c axis of (V, Mo)_5_Si_3_ which is above 10 × 10^−6^ K^−1^ [26] compared to the adjacent phases. However, the crack does not propagate into the substrate or cause coating delamination.

**Figure 5:**
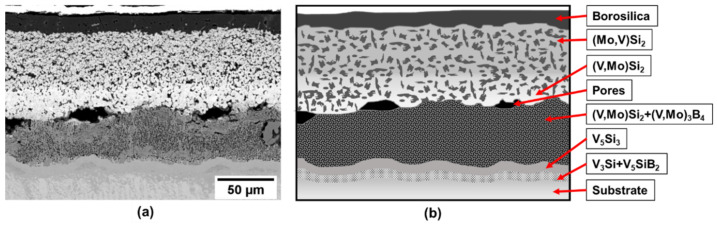
(**a**) Coating structure on a V-9Si-5B alloy after exposure at 1100 °C for 10 h and (**b**) schematic illustration of the coating structure.

The coating structure after exposure at 1100 °C for 10 h is presented in Figure 5.

**Figure 6:**
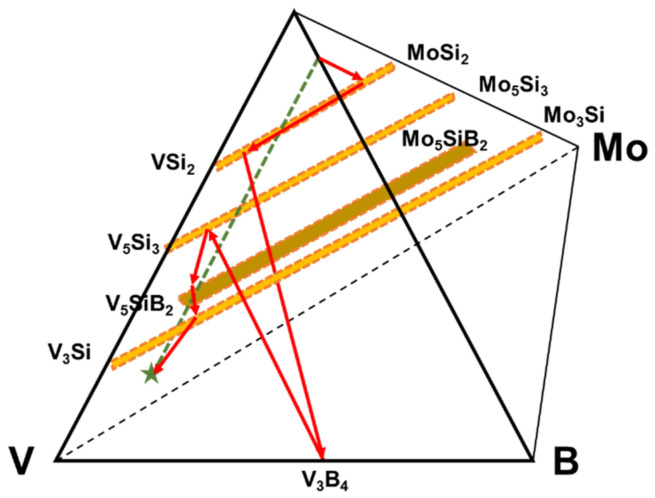
Illustration of the diffusion pathway on the V-9Si-5B substrate based upon the coating structure in Figure 5.

From the coating phase sequencing Figure 6 shows the projected diffusion pathway between the Si-B edge and the alloy substrate. In this case the diffusion pathway starts with the formation of the (Mo, V)Si_2_ phase that is enriched in V to the (V, Mo)Si_2_ phase as in the pure V case, but changes with the formation of the V_3_B_4_ phase implying a greater B flux than that observed for the pure vanadium substrate. The pathway then crosses the dotted mass conservation line with the phase sequence of (V, Mo)_5_Si_3_, (V, Mo)_5_SiB_2_ and (V, Mo)_3_Si phases before reaching the alloy substrate. The resultant coating does show good oxidation resistance and a closed borosilica layer on the surface. While the inner coating layers show also the absence of cracking which indicates mechanical compatibility to the V-9Si-5B substrate, the outer layers are rather porous and less dense which is attributed to a porous Mo pre-coating resulting from the spray coats.

## 4. Summary and Conclusions

The pack-cementation process by silicon and boron co-deposition is known to produce oxidation resistant coatings on various refractory based alloys and cermets and is able to prevent those materials from catastrophic oxidation failure by the “pesting” phenomenon [27,28]. In the current study, the coating strategy is combined with a novel and promising class of low-density, high temperature alloys from the V-Si-B system.

The present investigations identified the deposition of the Mo pre-coating as a key-point to achieve an adhering and protective Mo-Si-B coating on V-rich V-Si-B alloys. A successful powder pack process to implement Si and B strongly depends on a dense, pore-free Mo pre-coating that can be achieved by adjusting the sintering conditions after the Mo deposition, i.e., after the spray coating. Similarly, a further evaluation of the coated alloys at other temperatures and conditions is necessary. However, even without further optimization the coating application has demonstrated excellent coverage and oxidation resistance that now enables applications for the new class of V-Si-B alloys for high temperature operation.

## Figures and Tables

**Table 1:** Crystallographic data used for EBSD phase identification.

Phase	Lattice Parameter			Space Group	Database
	a	b	c		
V	3.03 Å	3.03 Å	3.03 Å	229	NSD
V_3_Si	4.72 Å	4.72 Å	4.72 Å	223	NSD
V_3_B_2_	5.75 Å	5.75 Å	3.04 Å	127	ICSD
V_5_SiB_2_	5.81 Å	5.81 Å	10.79 Å	140	ICSD
VB	3.10 Å	8.17 Å	2.98 Å	63	ICSD
V_5_Si_3_	7.14 Å	7.14 Å	4.84 Å	193	NSD
VSi_2_	4.57 Å	4.57 Å	6.37 Å	180	NSD
